# Circulating anti-hypothalamus antibodies in celiac patients: tissue transglutaminase friend or foe?

**DOI:** 10.1007/s12026-023-09394-0

**Published:** 2023-05-23

**Authors:** Erika Iervasi, Antonella Strangio, Luigi Greco, Renata Auricchio, Daniele Saverino

**Affiliations:** 1 Department of Experimental Medicine, University of Genoa, Via De Toni, 14, 16132 Genova, Italy; 2 Ospedale Policlinico San Martino, Largo Rosanna Benzi, 10, 16132 Genova, Italy; 3 Department of Translational Medical Science, University of Naples Federico II, Corso Umberto I, 40, 80138 Napoli, Italy; 4 European Laboratory for Food-Induced disease (ELFID), University of Naples Federico II, Via Pansini, 5, 80131 Napoli, Italy

**Keywords:** Active CD; Free-gluten CD; Anti-hypothalamus autoantibodies; Anti-tTG autoantibodies; Mucosal damage; Ghrelin

## Abstract

Celiac disease (CD) is an autoimmune disease with inflammatory characteristics, having a condition of chronic malabsorption, affecting approximately 1% of the population at any age. In recent years, a concrete correlation between eating disorders and CD has emerged. Hypothalamus plays a central role in determining eating behaviour, regulating appetite and, consequently, food intake. One hundred and ten sera from celiac patients (40 active and 70 following a gluten-free diet) were tested for the presence of autoantibodies against primate hypothalamic periventricular neurons by immunofluorescence and by a home-made ELISA assay. In addition, ghrelin was measured by ELISA. As control, 45 blood serums from healthy age matched were analysed. Among active CD, all patients resulted positive for anti-hypothalamus autoantibodies and sera showed significantly higher levels of ghrelin. All of the free-gluten CD were negative for anti-hypothalamus autoantibodies and had low levels of ghrelin, as well as healthy controls. Of interest, anti-hypothalamic autoantibodies directly correlate with anti-tTG amounts and with mucosal damage. In addition, competition assays with recombinant tTG showed a drastically reduction of anti-hypothalamic serum reactivity. Finally, ghrelin levels are increased in CD patients and correlated with anti-tTG autoantibodies and anti-hypothalamus autoantibodies. This study demonstrates for the first time the presence of anti-hypothalamus antibodies and their correlation with the severity of the CD. It also allows us to hypothesize the role of tTG as a putative autoantigen expressed by hypothalamic neurons.

## Introduction

Celiac disease (CD) is a common autoimmune-inflammatory condition, affecting about 1% people (65% women, 35% men) at any age [1]. CD is characterized by gluten-induced systemic signs: (1) presence of specific antibodies (autoantibodies of the IgA and/or IgG class against tissue transglutaminase, tTG, endomysium, and deamidated gliadin peptides (DGPs)); (2) specific human leukocyte antigen type (HLA-DQA1 and DQB1 alleles coding for the disease-associated DQ2 or DQ8 heterodimers); and (3) characteristic histological changes in the duodenum (flattened small intestinal mucosa with villous atrophy, crypt cell hyperplasia, and increased number of intraepithelial lymphocytes, resulting in nutrient malabsorption of variable degree) [1–3]. At the onset, patients may present typical manifestation (diarrhea, weight loss), may be oligosymptomatic, or may present atypical symptoms such as headache and psychiatric disturbances [4], including eating disorders (EDs) [5]. Thus, it seems that among the numerous extraintestinal manifestations of CD, psychological comorbidity should be included [6–8]. So, the association between ED and CD deserves attention. In this regard, undiagnosed CD could predispose to mental and behavioural disorders, and great attention should be maintained to exclude this illness in subjects with CD [9–12]. Literature is showing evidence that the prevalence of ED is about twice that of teenage girls with type 1 diabetes compared to age-matched controls [13]. In addition, this condition could be of particular concern in patients with concurrent diabetes and CD: as it seems that deliberate insulin under-dosing or omission is a common strategy for weight loss in diabetic women with eating disorders [14]. Therefore, the presence of an ED should also be carefully studied in people with active CD. In this context, as appetite is controlled by regulatory peptides acting on specific neuronal receptors in the hypothalamus, we set up this study to evaluate the presence of autoantibodies directed to receptors expressed by hypothalamic cells.

In addition, few studies analysed the role ghrelin in CD. Ghrelin is a gastrointestinal hormone that is primarily secreted by the stomach and duodenum [15, 16]. It has orexigenic action stimulating appetite, reducing fat utilisation, producing adiposity, and inducing hyperglycemia [16]. Ghrelin orexigenic effects can prevail the anorectic action of leptin [15]. Ghrelin and leptin would have complementary activities in a single regulatory system which would have developed to inform the central nervous system about the state of energy balance [15]. Because CD patients have intestinal histopathologic alterations and thus malnutrition and/or growth failure, alterations in serum ghrelin levels in CD can be hypothesized.

Thus, the main objective of this study was to verify the presence of anti-hypothalamus autoantibodies (where neurons related to eating habits dwell) and ghrelin levels in sera from CD patients.

## Materials and methods

### Patients’ characteristics

Blood samples were collected from 110 CD patients (Table 1), all of whom provided written informed consent. The research was approved by the Ethics Committee of the School of Medicine, University of Naples “Federico II,” Italy, and was in accordance with the principles of the Helsinki II Declaration. The diagnosis was based on ESPGHAN criteria [Working Group of ESPGAHN, 1990], and modified Marsh classification was used (T0, normal lymphocyte infiltration and villous atrophy; T1, more than 30% increased intraepithelial lymphocyte infiltration, lymphocytic enteritis; T2, T1 with crypt hyperplasia; T3a, T2 with partial villous atrophy; T3b, T2 with subtotal villous atrophy; T3c, T2 with total villous atrophy) (Table 2) [18]. Anti-tissue transglutaminase IgA (anti-tTG IgA) was determined by commercial enzyme-linked immunosorbent assay (ELISA) using microplates coated with recombinant human antigen (Eurospital Diagnostics, Trieste, Italy), as previously described [17].

**Table 1:** Demographic characteristics of study groups

	Age (years)	Mucosal damage (Marsh, number)	anti-tTG IgA autoantibodies (ng/ml)
Healthy blood donors (*n* = 45)	19–45 30 female, 15 male	Not applicable	Not detectable
CD total (*n* = 110) Untreated (*n* = 40) Treated (*n* = 70)	1–41 85 female, 35 male	T0, *n* = 55 T1, *n* = 15 T2, *n* = 16 T3, *n* = 24	6.9 ± 2.2
	1–41 21 female, 19 male	T2, *n* = 16 T3, *n* = 24	9.1–26
	18–48 64 female, 16 male	T0, *n* = 55 T1, *n* = 15	0.1–8.5

Untreated: CD patients at the diagnosis; Treated: CD patients following a gluten-free diet

**Table 2:** Modified Marsh classification of histologic findings in celiac disease used for patients’ classification [17]

Marsh type	IEL/100 enterocytes-jejunum	IEL/100 enterocytes-duodenum	Cripthyperplasia	Villi
0	< 40	< 30	Normal	Normal
1	> 40	> 30	Normal	Normal
2	> 40	> 30	Increased	Normal
3a	> 40	> 30	Increased	Mild atrophy
3b	> 40	> 30	Increased	Marked atrophy
3c	> 40	> 30	Increased	Complete atrophy

IEL/100 enterocytes-jejunum: intraepithelial lymphocytes per 100 enterocytes. Marsh type 0: normal; potential CD; CD highly unlikely. Marsh type 1: seen in patients on gluten free diet (suggesting minimal amounts of gluten or gliadin are being ingested); potential CD; patients with dermatitis herpetiformis; family members of CD patients, not specific, may be seen in infections. Marsh type 2: very rare, seen occasionally in dermatitis herpetiformis. Marsh type 3: spectrum of changes seen in symptomatic CD

Forty patients were in active phase of CD (age range 0.8–51): 12 of them were adult (i.e. > 18 years old), and the large majority (10/12) were poorly symptomatic, presenting anaemia or reduced bone mineral density. Seventy patients were in remission under gluten-free diet (age range 16–48). Remission of disease was documented by disappearance of serum autoantibodies and clinical manifestations. The control groups consisted of 45 healthy volunteer blood donors (age range 19–45). Sera were stored frozen until the use, and freezing and thawing were avoided.

Finally, none of the enrolled CD patients as well as healthy controls was suffering from eating disorders and/or psychological symptoms or other autoimmune comorbidities.

### Immunofluorescence

The detection of anti-hypothalamic autoantibodies was performed with the IIF method using kits provided free of charge by Euroimmun AG (Lubeck, Germany) [19, 20]. In detail, each sample was analysed at a serum dilution of 1:10 on two different sections of primate hypothalamus. Fluorescent anti-human Ig conjugate was used (F(ab′)-goat anti-human IgG, Alexa Fluor™ 488, Thermo Fischer Scientific, Monza, Italy). A positive and a negative control serum were analysed in each analytical session. The intensity of the fluorescence was determined by two different readers blind to subject characteristic, and a scale from 0 to 3 was assigned (0 no reaction, 1 weak or uncertain fluorescence, 2 moderate fluorescence, 3 high fluorescence). Samples scored positive if a 2 or 3 fluorescence reaction was observed [20].

### Immunofluorescence assay for transglutaminase interference

Immunofluorescence interference tests were performed by slides of frozen primate periventricular hypothalamus (Euroimmun, Lubeck, Germany), as previously described. In order to understand the role of tTG as potential molecule recognized by autoantibodies present in CD sera, transglutaminase previously depleted sera were used. To this end, 40 sera from active CD and 40 from gluten-free diet CD sera were incubated for 3 h at room temperature on ELISA plates coated with human tissue transglutaminase recombinant soluble molecule (1 mg/ml) (EMELCA Bioscience, Antwerp, The Netherlands). This procedure was repeated twice before utilising these sera in immunofluorescence assays. In addition, at the end of the second depletion cycle, sera were analysed by a specific ELISA test to confirm negativisation of anti-tTG IgA (Eurospital Diagnostics, Trieste, Italy). For all samples, results were under the limit of the test’s detection (data not shown).

After depletion, sera were serially diluted from 1:10 to 1:80 in PBS (pH 7.3), as necessary, and immunofluorescence tests were performed. After incubating for 30 min at room temperature, fluorescent anti-human immunoglobulin class G (IgG) conjugate was used, as previously described.

As control, sera without tTG depletion were used.

### ELISA

Specific ELISA kits were used for measuring serum ghrelin levels (EMELCA Bioscience, Breda, the Netherlands), according to the manufacturer’s protocol. Each sample was diluted 1:10 and tested in triplicate. Deviation between triplicates was < 10% for any reported value. The lowest sensitivity threshold was 0.31 ng/ml. The analytical response was linear approximately between 0.450 and 2.250 of absorbance values (corresponding to 0.31–20 ng/ml) as assessed by serial dilution test using a strongly positive serum (data not shown). For samples with serum ghrelin concentration higher than 20 ng/ml, the ELISA tests were repeated using a greater dilution factor (1:100).

### Statistics

Statistical analysis was performed by using the Mann–Whitney *U* test for comparison of ghrelin and anti-tTG levels. Spearman correlation analysis was used to evaluate the relation between ghrelin or anti-tTG IgA levels, and anti-hypothalamic antibodies. A *p* value of less than 0.05 was considered statistically significant. All the analyses were performed by using the GraphPad Prism software 6.0 (GraphPad Software Inc., CA, USA).

## Results

### Anti-hypothalamic autoantibodies can be detected in sera from active CD patients

**Fig. 1:**
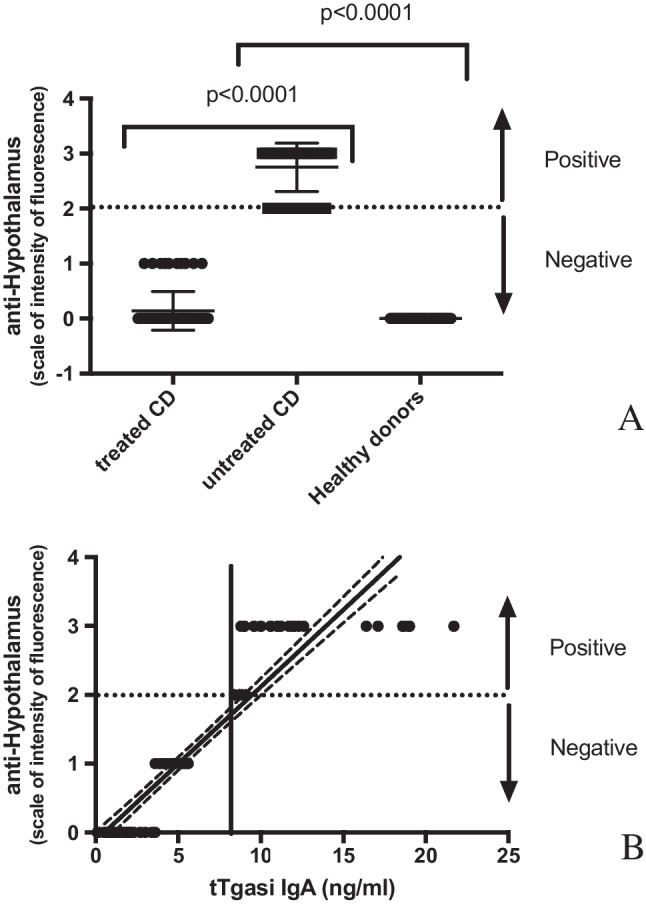
Evaluation of the presence of anti-hypothalamus IgG autoantibodies in CD patients and in healthy donors. ***A*** Anti-hypothalamus autoantibodies are present in sera from active CD patients, but they are not in gluten-free diet CD patients (*p <* 0.0001), neither in healthy donors (*p <* 0.0001). Anti-hypothalamic autoantibodies were detected by IIF method. A positive and a negative control serum were analysed in each analytical session. The intensity of the fluorescence was determined by two different readers blind to subject characteristic and a scale from 0 to 3 was assigned (0 no reaction, 1 weak or uncertain fluorescence, 2 moderate fluorescence, 3 high fluorescence). Samples scored positive if a 2 or 3 fluorescence reaction was observed. **B** A correlation of tTG-IgA autoantibodies vs. anti-hypothalamus IgG can be depicted. Untreated and treated CD patients are characterised by positive (> 9 ng/ml) and negative (< 9 ng/ml) anti-tTG autoantibody amounts respectively. A vertical line represents the cut-off value of 7 ng/ml for anti-tTG IgA autoantibodies (as suggested by the manufacturer’s protocols). Dashed lines indicate the 95% confidence interval of the best-fit line (continuous lines)

One hundred ten sera from celiac patients were tested (40 active and 70 following a gluten-free diet) for the presence of hypothalamic autoantibodies. In the group of active CD, all patients resulted positive for anti-hypothalamus autoantibodies (in detail, 11 showed moderate fluorescence and 29 high fluorescence). All of the free-gluten CD were negative (58 no reaction, 12 weak or uncertain fluorescence) as well as healthy controls (in depth, 44 no reaction, 1 weak or uncertain fluorescence), with a statistically significant difference between the two groups of CD patients (*p* < 0.001) (Fig. 1A). Finally, the control group of 45 healthy volunteers showed undetectable anti-hypothalamic autoantibody.

### Anti-hypothalamic autoantibodies directly correlate with anti-tTG amounts

Active and treated CD are characterised respectively by negative (< 9 ng/ml) and positive (> 9 ng/ml) anti-tTG IgA autoantibody levels. Thus, we analysed the possible relationship between the anti-tTG IgA values of the sera of CD and the presence of anti-hypothalamus antibodies (IgG). As shown in Fig. 1B, a significant positive correlation between the presence of anti-hypothalamus antibodies and the amount of anti-tTG autoantibodies is apparent (*R* = 0.6697).

### Human recombinant tTG are able to interfere with autoantibody assays

**Fig. 2:**
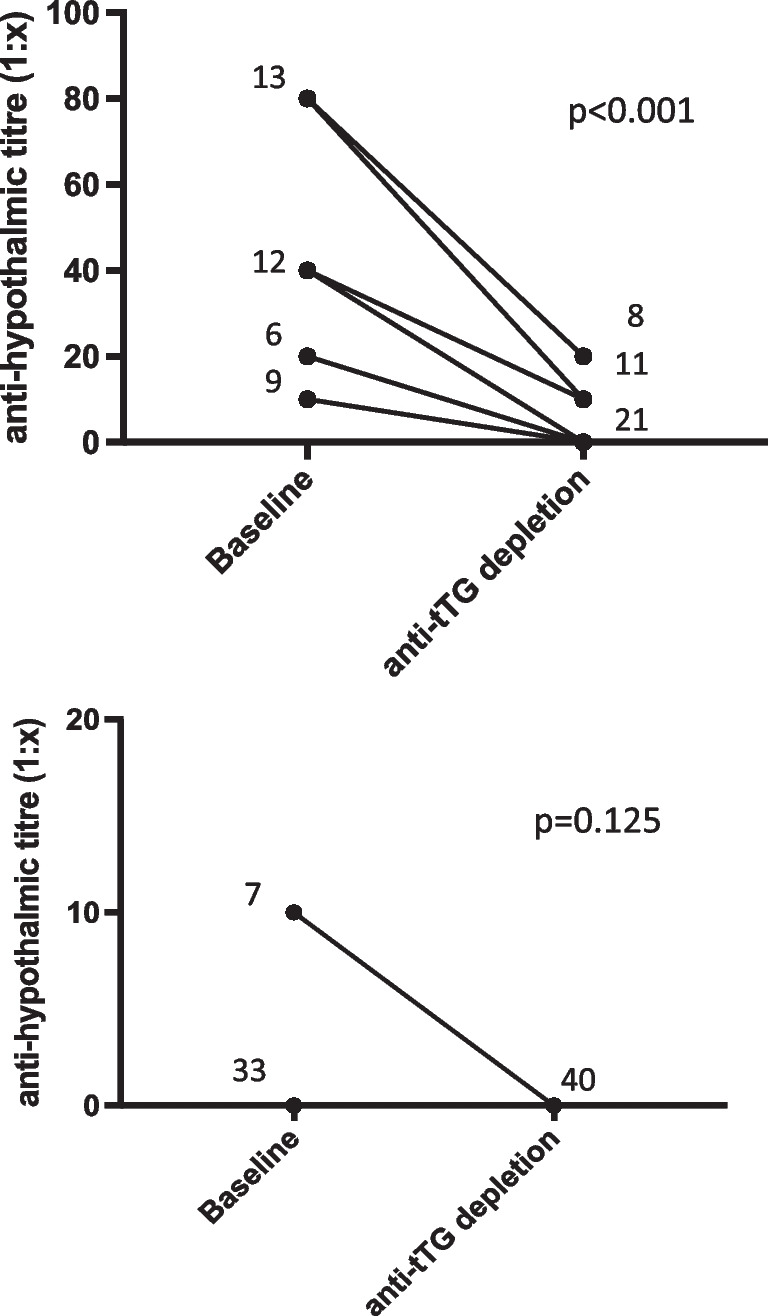
tTG molecule is able to interfere with autoantibody binding. As tTG molecules are expressed by different brain tissues, and tTG is thought to play a role in oxidative damage, experiments were set up to investigate whether they may interfere with the binding of reactive autoantibodies to hypothalamic tissues. Fourty sera from active (untreated CD) and 40 from gluten-free diet (treated CD) sera were titred before and after tTG depletion. Numbers of sera patients for each dilution are shown

As shown in Table 3 and in Fig. 2, anti-tTG depletion of sera with human recombinant tTG inhibits the reaction of serum autoantibodies to hypothalamic cells in a different way. This competition effect is measurable as strong a reduction of the positivity titre. However, immunofluorescence staining does not become totally negative (Table 3 and Fig. 2).

**Table 3:** Immunofluorescence titre of autoantibody reactivity on hypothalamic cells from CD serum, before (baseline) and after depletion (+tTG) by recombinant human tTG

Untreated CD			Treated CD		
	Baseline (1:*x*)	+tTG (1:*x*)		Baseline (1:*x*)	+tTG (1:*x*)
1	10	0	1	0	0
2	10	0	2	0	0
3	10	0	3	0	0
4	10	0	4	0	0
5	10	0	5	0	0
6	10	0	6	0	0
7	10	0	7	0	0
8	10	0	8	0	0
9	10	0	9	0	0
10	20	0	10	0	0
11	20	0	11	0	0
12	20	0	12	0	0
13	20	10	13	0	0
14	20	0	14	0	0
15	20	0	15	0	0
16	40	0	16	0	0
17	40	0	17	0	0
18	40	0	18	0	0
19	40	0	19	0	0
20	40	0	20	0	0
21	40	0	21	0	0
22	40	10	22	0	0
23	40	10	23	0	0
24	40	10	24	0	0
25	40	0	25	0	0
26	40	10	26	0	0
27	40	10	27	0	0
28	80	10	28	0	0
29	80	10	29	0	0
30	80	10	30	0	0
31	80	10	31	0	0
32	80	20	32	0	0
33	80	20	33	0	0
34	80	20	34	10	0
35	80	10	35	10	0
36	80	20	36	10	0
37	80	20	37	10	0
38	80	20	38	10	0
39	80	20	39	10	0
40	80	20	40	10	0

### Ghrelin levels are increased in CD patients and correlated with anti-tTG autoantibodies

**Fig. 3:**
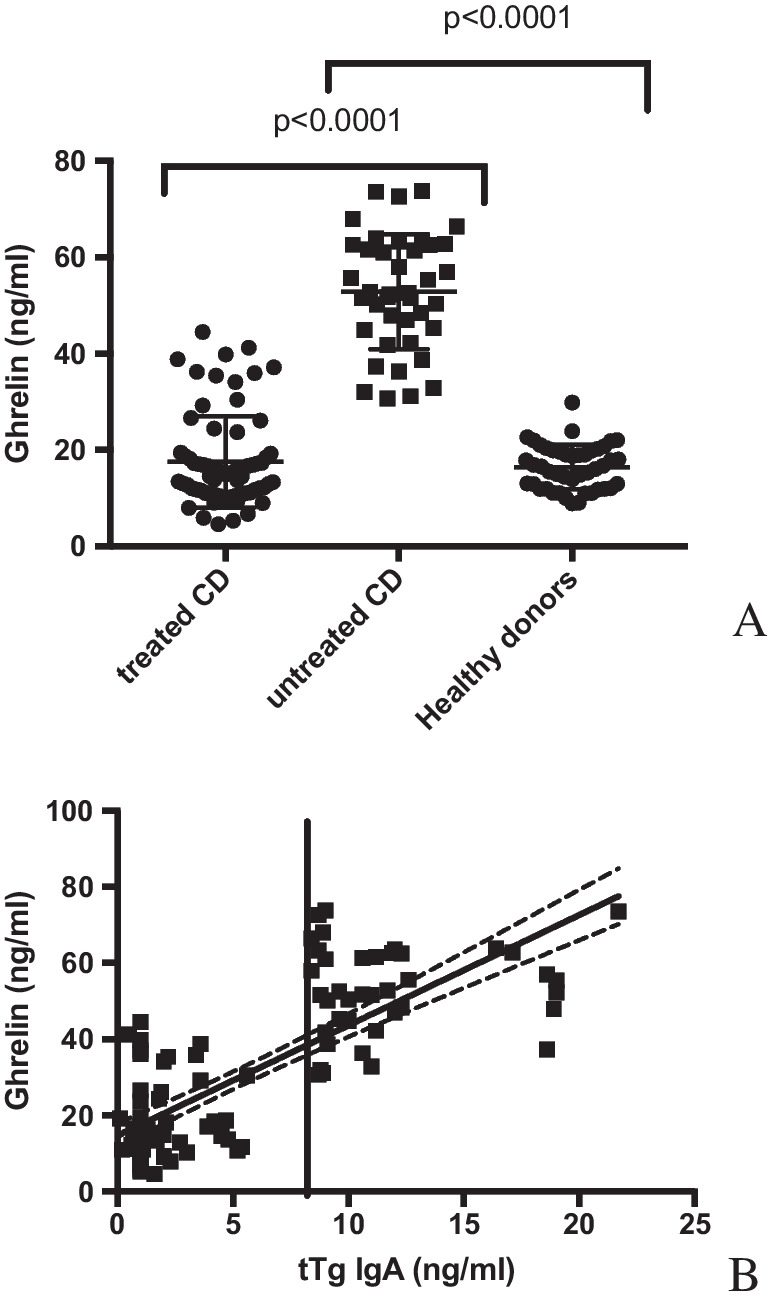
Evaluation of the presence of ghrelin in sera of CD patients and healthy donors. **A** Serum amount of ghrelin was measured by a specific ELISA test. As can be depicted, a gluten-free diet (treated CD) significantly decreases the total serum ghrelin concentration measured during the active phase of CD (untreated CD). Ghrelin levels in treated CD patients and healthy donor do not significantly differ. ***B*** A correlation of tTG autoantibodies vs. ghrelin serum amounts can be depicted. CD population can be clustered in three groups: CD patients following a gluten-free diet are anti-tTG negative and express a lower amount of ghrelin. Among active CD, two groups can be highlighted: those that were characterised by an intermediate level of both anti-tTG autoantibodies and ghrelin and that showed high levels of both anti-tTG autoantibodies and ghrelin. A vertical line represents the cut-off value of 7 ng/ml for anti-tTG IgA autoantibodies (as suggested by the manufacturer’s protocols). Dashed lines indicate the 95% confidence interval of the best-fit line (continuous lines)

It has been shown that orexigenic ghrelin stimulates growth hormone release, appetite, adipogenesis and gut motility and inhibits expression of pro-inflammatory cytokines [21]. For this reason, serum ghrelin levels were evaluated. Figure 3A compares ghrelin levels in CD patients. Active CD patients show significantly higher levels of ghrelin (range 30.72–73.80 ng/ml, mean 52.83 ng/ml, st. dev. 11.92), than treated patients (range 4.58–44.52 ng/ml, mean 17.52 ng/ml, st. dev. 9.51), *p* < 0.0001. Finally, in the control group of 45 healthy volunteers, serum ghrelin levels were similar to those of treated CD (range 8.91–29.82 ng/ml, mean 16.42 ng/ml, st. dev. 11.92).

Figure 3B highlights the correlation between the anti-tTG autoantibody values and the amounts of ghrelin in CD patients. Vertical line (tTG IgA = 7 ng/ml) indicates the lower positive amounts used for discriminate patients with active CD. As can be observed, CD population can be clustered in three groups: CD patients following a gluten-free diet are anti-tTG negative (< 9 ng/ml; 1.767 ng/ml mean, 1.359 st. dev.) and express a lower amount of ghrelin (< 44 ng/ml; 17.5 ng/ml mean, 9.575 st. dev.). A second group, among active CD, was characterised by an intermediate level of anti-tTG (from 9 to 15 ng/ml; 9.991 ng/ml mean, 1.51 st. dev.). The third group had high levels of anti-tTG (> 15 ng/ml; 18.69 ng/ml mean, 1.56 st. dev.); both these two groups were characterised by higher levels of ghrelin (from 31 to 73.80 ng/ml, 51.97 ng/ml mean, 12.15 st. dev. and from 37.28 to 73.63 ng/ml, mean 56.28 ng/ml, st. dev. 11.01 respectively). All of these results suggest that anti-tTG autoantibodies and ghrelin production are directly correlated (Spearman *r* = 0.713, *p* < 0.001).

### Ghrelin levels correlate with anti-hypothalamus autoantibodies

**Fig. 4:**
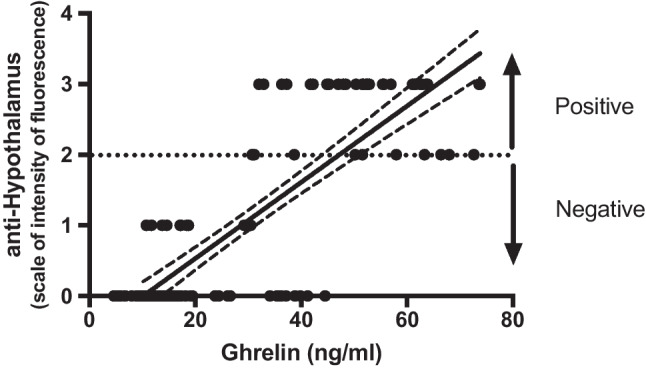
A significant positive correlation among anti-hypothalamus autoantibodies vs. ghrelin exists. The quantitative evaluation of ghrelin and the qualitative presence of anti-hypothalamus antibodies detected in the total CD population, independently by diet show a positive correlation

Finally, a comparison between anti-hypothalamus autoantibodies and ghrelin levels was performed. Figure 4 shows data of the quantitative evaluation of ghrelin and the qualitative presence of anti-hypothalamus antibodies detected in the total CD population, independently by diet. Ghrelin amount range was from 4.58 to 73.8 ng/ml (30.36 ng/ml mean, 19.98 st. dev.), whereas anti-hypothalamus autoantibodies could be clustered in two groups: positive (11 showed moderate, and 29 high fluorescence) and negative (58 no reaction, 12 weak or uncertain fluorescence). A positive correlation between these two parameters is evident (Spearman *r* = 0.7628, *p* < 0.001). When patients were divided in active CD and in gluten-free diet (treated) CD, the first showed a higher level of both ghrelin and anti-hypothalamus autoantibodies (range 30.72–73.80 ng/ml, mean 52.42 ng/ml, and moderate/high fluorescence intensity respectively). On the other hand, gluten-free diet CD showed lower ghrelin levels (range 4.58–44.52 ng/ml, mean 14.88 ng/ml) and no fluorescence on hypothalamus specimens. Table 4 summarises results from this study.

**Table 4:** Summary of the results obtained in this study

	Free diet			No-gluten diet		
	tTGasi (ng/ml)	Anti-hypothalamus mAb	Ghrelin (ng/ml)	tTGasi (ng/ml)	Anti-hypothalamus mAb	Ghrelin (ng/ml)
Number of values	40	40	40	70	70	70
Minimum	7.4	2	30.72	0	0	4.58
25% Percentile	8.8	2.25	44.98	1	0	11.02
Median	10.6	3	52.42	1	0	15.04
75% Percentile	12.3	3	62.6	2.02	0	18.82
Maximum	21.7	3	73.8	5.6	1	44.52
Mean	11.73	2.75	52.83	1.74	0.14	17.52
Std. deviation	3.820	0.438	11.920	1.372	0.352	9.507
Std. error of mean	0.604	0.069	1.885	0.163	0,042	1.136
Lower 95% CI of mean	10.5	2.61	49.01	1.409	0.056	15.26
Upper 95% CI of mean	12.95	2.89	56.64	2.063	0.227	19.79

The intensity of the fluorescence of anti-hypothalamus autoantibodies was determined by two different readers blind to subject characteristic, and a scale from 0 to 3 was assigned (0 no reaction, 1 weak or uncertain fluorescence, 2 moderate fluorescence, 3 high fluorescence). Samples scored positive if a 2 or 3 fluorescence reaction was observed

### Anti-hypothalamus autoantibodies and ghrelin levels correlate with mucosal damage

**Fig. 5:**
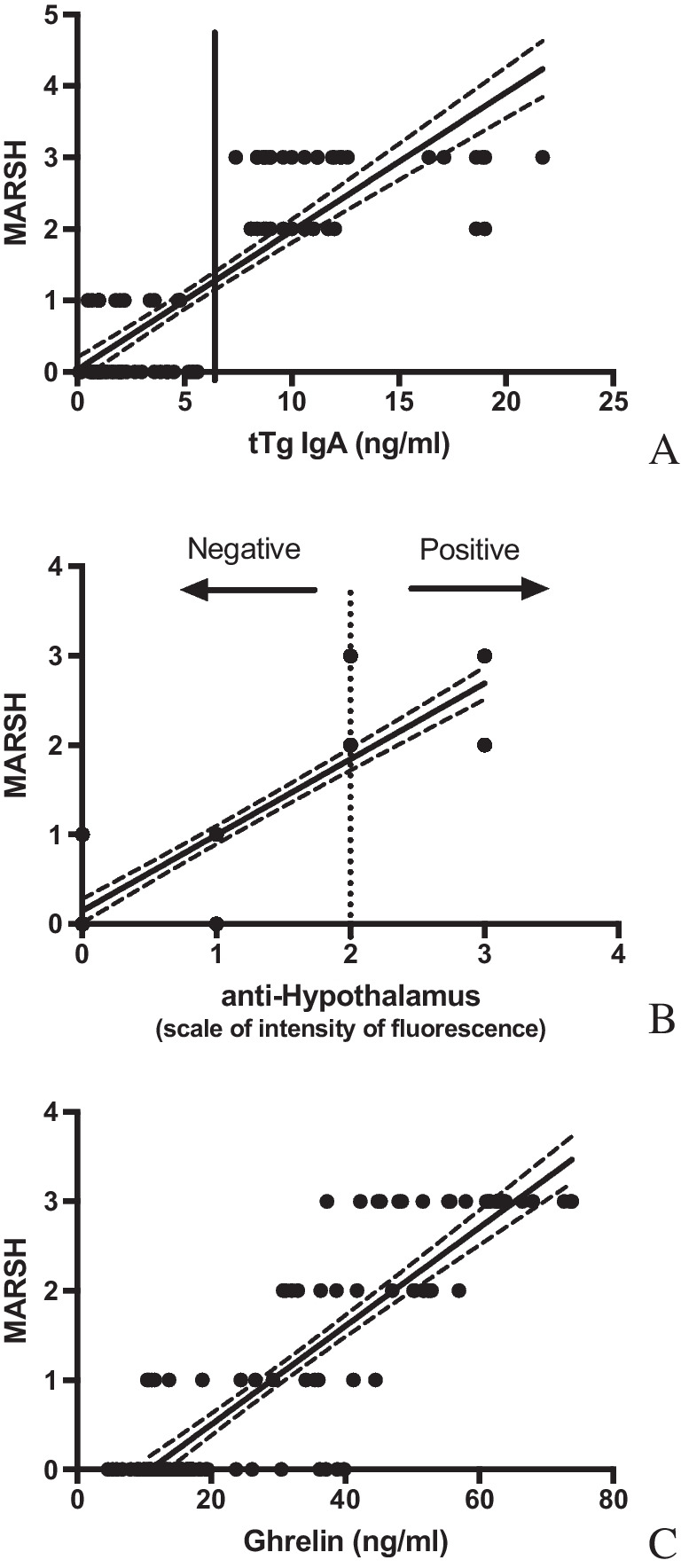
Anti-hypothalamus autoantibodies and ghrelin levels correlate with mucosal damage. **A** As expected, anti-tTG autoantibody values correlated mucosal damage (classified according to the modified Marsh). **B** A significant positive correlation was observed when anti-hypothalamus autoantibodies were compared to mucosal damage (modified Marsh classification). **C** Finally, it seems ghrelin production reflects the local mucosal damage

Noteworthy, a significant positive correlation was observed when anti-hypothalamus autoantibodies or serum levels of ghrelin were compared to histological grading classified according to the modified Marsh classification (Spearman *r* = 0.8456, *p* < 0.0001, and *r* = 0.8145, *p* < 0.0001 respectively). Thus, it seems that anti-hypothalamus autoantibodies (Fig. 5B) and ghrelin (Fig. 5C) production reflect the local mucosal damage. As expected, anti-tTG autoantibody values correlated mucosal damage (Spearman *r* = 0.7845, *p* < 0.0001) (Fig. 5A).

## Discussion

Autoimmune diseases result of alterations of mechanisms responsible for tolerance to the “self.” Among autoimmune diseases, those affecting the intestinal tract, such as celiac disease (CD), Crohn’s disease and ulcerative colitis, are supposed to be related with eating disorders, such as anorexia nervosa, or bulimia nervosa. Thus, this relationship should not be underestimated. In CD, especially children, anorexia symptoms develop absolutely early and require priority attention. In this context, anorexia symptoms appear early, and they can also anticipate any other specific symptoms, such as weight loss or diarrhoea.

From a genomic point of view, the first significant association between anorexia nervosa (AN) and autoimmune diseases has been identified in a region already known for its involvement in autoimmune diseases including type 1 diabetes and arthritis [22–24]. More recently, Mårild et al. [10] demonstrate an association between AN and celiac disease, both before and after diagnosis of celiac disease. On the other hand, association of ED with autoimmunity is rather complex to be analysed as it is necessary to take into account the influence of numerous genetic variants that can collaborate in an additive way with environmental factors, which will act on phenotypic expression [23].

Current evidence suggests that several biological factors’ modification, such as adipokines [25, 26], estrogen levels and alteration of intestinal microbiota [25, 27], can affect immune function in ED. Finally, the presence of autoantibodies against appetite-regulating neuropeptides is emerging as key player in the peptidergic mechanisms controlling feeding behaviours [28–30].

From the anatomical point of view, hypothalamus plays a central role in eating behaviour and contains the main nervous centre capable of regulating appetite. Leptin, insulin, ghrelin and other hormones are able to act on neurons within the arcuate nucleus, antagonistically regulating the control of energy intake. These neurons express orexigenic receptors (orexigenic agouti-related peptide (AgRP) and neuropeptideY (NPY)) or anorexic peptides (α-melanocyte stimulating hormone (α-MSH) and the regulatory transcripts cocaine and amphetamine (CART)) [21].

It is well known that ghrelin is able to regulate the release of growth hormone (GH), appetite, adipogenesis and intestinal motility and inhibits the expression of pro-inflammatory cytokines. It has also been shown that the administration of ghrelin, also to man, induces the retention of lean body mass and the decrease of circulating pro-inflammatory cytokines, but not inflammation of the mucous membranes [31–34].

Given the central role of hypothalamic neurons in regulating the perception of hunger/appetite, this study aimed to highlight if an autoantibody response against these neurons in CD patients exists, and if a relationship among these autoantibodies and serological/histopathological parameters can be detected. Finally, evaluation of the amount of serum ghrelin was evaluated.

This study shows for the first time that anti-hypothalamus antibodies are present in serum of CD patients who are not on a gluten-free diet. Of interest, anti-hypothalamus antibodies become negative after gluten-free diet, or at least not detectable as in healthy controls. In addition, the concentration of these autoantibodies correlates with the amount of anti-tTG and with the mucosal damage. Therefore, since the hypothalamic arcuate nucleus is directly involved in appetite control (both in energy intake and in its consumption), an active role of autoantibodies directly against hypothalamus in regulating feeding behaviours should be speculated. In addition, it could be hypothesized that these antibodies are directed against tTG. In fact, this molecule is ubiquitous and also expressed by neurons in the hypothalamus. It has been demonstrated that neurons in the medial preoptic, in the lateral hypothalamic area and in the periventricular nuclei express constitutively tTG [34, 35]. Thus, tTG expression in different brain areas and in distinct classes of neurons suggest various brain functions. In addition, tTG was found in the neuronal soma, dendrites, axon shafts and axon endings, which generally associate with the mitochondrial outer wall [34, 35]. There is evidence for increased damage, associated with mitochondrial dysfunction of macromolecules in several autoimmune diseases [36]. In this study, we suggest tTG as possible antigen of anti-hypothalamus antibodies present in the serum of active CD patients. However, results from depletion experiments indicated that, although it is the main target, in addition to tTG, other unknown target molecules for autoantibodies present in active CD serum may exist. Thus, further studies are needed: to verify the effective specificity of the anti-hypothalamus antibodies highlighted in this study and to understand the effective role of these autoantibodies in the association between anorexia and CD patients in the acute phase.

In addition, untreated CD patients showed significant increase of serum ghrelin levels as compared to the levels in CD following a free-gluten diet (treated CD). Of interest, ghrelin levels seem to correlate with anti-tTG levels and mucosal damage. Results from previous studies are not unique [37–43]. Some authors have shown significant increase of the ghrelin levels in untreated CD patients that returned to normal during a gluten-free diet [37]. Other authors reported the same ghrelin concentrations in untreated CD adults and in controls that significantly decreased during a gluten-free diet [38]. Other yet authors have shown ghrelin levels in untreated CD adults fivefold higher than those in controls [39]. Although the reasons for these discrepancies are unclear, we observed that there is a positive direct correlation with anti-tTG levels and fluorescence intensity of anti-hypothalamus staining in untreated CD patients. These findings suggest that increased ghrelin levels in CD patients are due to a specific action of the immune system within the pathogenesis of this disease. Also, because ghrelin returns to levels almost no measurable after gluten free-diet, as it happens in normal subjects.

## Conclusions

In summary, some important findings have been achieved. The presence of anti-hypothalamic antibody elevation was not age- or sex-dependent, but conditional to gluten intake. And then, it correlates with the title of anti-tTG autoantibodies and with mucosal damage. Ghrelin levels were not significantly different between the sexes or ages, but its production was influenced by the gluten-free diet. And even more interesting, ghrelin levels were correlated with the presence of anti-hypothalamus antibodies.

This study demonstrates for the first time the presence of anti-hypothalamus antibodies, tTG as putative autoantigen, and the correlation with the severity of the CD. However, it would be interesting to continue the study, analysing the same parameters in CD and eating disorders in concomitant patients.

## Abbreviations

CD: Celiac disease
ED: Eating disorders
tTG: Tissue transglutaminase

## Declarations

### Guarantor of the article

Daniele Saverino, MD PhD.
